# Incidence of and Risk Factors for Prolonged Intensive Care Unit Stay After Open Heart Surgery Among Elderly Patients

**DOI:** 10.7759/cureus.31602

**Published:** 2022-11-17

**Authors:** Fatma İrem Yeşiler, Nursultan Akmatov, Oktom Nurumbetova, Deniz Sarp Beyazpınar, Helin Şahintürk, Ender Gedik, Pınar Zeyneloğlu

**Affiliations:** 1 Department of Anesthesiology and Critical Care Unit, Baskent University Faculty of Medicine, Ankara, TUR; 2 Department of Cardiovascular Surgery, Baskent University Faculty of Medicine, Ankara, TUR; 3 Department of Anesthesiology and Critical Care Unit, Başkent University Faculty of Medicine, Ankara, TUR

**Keywords:** postoperative complications, postoperative mechanical ventilation, cardiovascular disease, prolonged intensive care stay, prolonged stay, intensive care unit, open heart surgery, elderly patient

## Abstract

Objective: Open heart surgery (OHS) is frequently performed on elderly patients. We aimed to investigate the risk factors associated with prolonged intensive care unit (ICU) stay in elderly patients undergoing open heart surgery.

Materials and Methods: Medical records of all patients ≥ 75 years who underwent OHS (coronary artery bypass grafting (CABG) and/or heart valve surgery) between June 1, 2013, and December 31, 2020, were retrospectively analyzed. Those staying in the ICU longer than five days were determined as prolonged ICU stay. Patients were divided into two groups, according to ICU stay <5 days and ≥5 days.

Results: Out of the 198 patients included in the study, 130 (65.7%) were male. Seventy patients (35.4%) had prolonged ICU stay. The mean age was higher in patients within the prolonged ICU stay group when compared to the other group (79.9±3.5 years vs.78.1±2.7 years, p<0.001). The patients who used statins and angiotensin-converting enzyme inhibitors (ACEi)/angiotensin receptor blockers (ARBs) in the preoperative period had a shorter ICU stay compared to those who did not (45% vs 31.4%, p=0.04; 57% vs 42.9%, p=0.03). The history of previous thoracic surgery (2.3% vs 10% p=0.03), emergency surgery (12.5% vs 24.5% p=0.04), and preoperative pacemaker usage (0.8% vs 7%, 1 p=0.01) were higher in the group of patients with prolonged ICU stay compared to the other group. Preoperative ejection fraction (EF)% (47.7±11.3 vs 51.1±8.8, p<0.001) and hemoglobin level (11.8±1.9 mg/dL vs 12.9±1.6, p<0.001) were lower in the group with prolonged ICU stay compared to the other group. Incidence of cardiac arrest (3.9% vs 15.7% p=0.006), presence of arrhythmia (16.4% vs 41.6%,p<0.001), frequency of pacemaker and intra-aortic balloon pump (IABP) usage (0 vs 10% p=0.002; 1.6% vs 8.6% p=0.02), and need for renal replacement therapy (3.1% vs 12.9%,p=0.02) were higher in the group with prolonged ICU stay compared to the other group. According to the logistic regression analysis; higher age (OR: 1.225, 95%CI 1.104-1.360, p<0.001), preoperative pacemaker usage (OR: 0.100, 95%CI 0.01-0.969, p<0.04), preoperative statin non-use (OR: 2.056, 95%CI 1.040-4.066, p<0.03) and preoperative low EF (OR: 0.947, 95%CI 0.915-0.981, p=0.002) were determined as independent risk factors for prolonged ICU stay.

Conclusion: The incidence of prolonged ICU stay after OHS among patients ≥75 years was 35.4% in our cohort. Higher age, preoperative pacemaker usage, preoperative statin non-use, and low preoperative EF were associated with prolonged ICU stay.

## Introduction

Open heart surgery (OHS) is one of the treatment options for cardiovascular diseases. OHSs mainly include coronary artery bypass graft surgery (CABG), valvular heart surgery (VHS), heart transplantation, and congenital heart surgery [1]. The aim of OHS in elderly patients is to eliminate the symptoms of cardiovascular diseases, restore cardiac function, and improve quality of life.

The number of patients undergoing OHS has gradually increased with the prolonged life expectancy and increasing medical developments. OHS is performed on elderly patients with more comorbidities and higher risk. As a result of the developments in anesthesia technique, surgical method, intensive care unit (ICU) follow-up and management, duration of postoperative mechanical ventilation, and length of ICU and hospital stay have decreased significantly. However, there are many patients who stay in ICU and need mechanical ventilation for a long time due to various complications in the postoperative period. These issues increase the economic burden and also prevent the usage of ICU equipment for other patients. It has been reported that prolonged ICU stay can be observed at approximately 19-45% after OHS [2-4].

Advanced age (65 years and over) is among the important risk factors of OHS. OHS operations, which have multisystemic effects, cause great trauma to the metabolism, whose reserves are already reduced due to older age. In the elderly patient group, the number of complications in the postoperative period increase, and the duration of mechanical ventilation and ICU stay are prolonged [4-6].

We aimed to retrospectively investigate the incidence of prolonged ICU stay and risk factors associated with prolonged ICU stay among ≥ 75 years old patients who underwent OHS (CABG and/or VHS) at our center.

This article was previously presented as a meeting abstract at the 21st National Intensive Care Congress on March 17-20, 2022.

## Materials and methods

This study was approved by the Başkent University Institutional Review Board (project no: KA22/112). Medical records of all patients ≥ 75 years who underwent OHS (CABG and/or VHS) between June 1, 2013, and December 31, 2020, were retrospectively analyzed. Patients were divided into two groups, according to those ICU stay <5 days and ≥5 days. If the length of ICU stay was ≥ 5 days, it was defined as prolonged ICU stay. We determined prolonged ICU stay according to our current clinical practice and previous studies [2,5,7,8].

There are different classifications that group elderly people by age. Some studies have classified elderly adults between the ages of 65 and 74 years as youngest-old, those between ages 75 and 84 years as middle-old, and those aged over 85 years as oldest-old [9]. According to this classification, we included middle-old and oldest-old patients in the study.

The primary goal of the study is to determine the risk factors associated with prolonged ICU stay after OHS in elderly patients ≥ 75 years. The secondary goal is to determine the incidence and complications of prolonged ICU stay.

Data collection

Preoperative, intraoperative, and postoperative data of the patients were obtained from electronic medical, anesthesia follow-up, and nursing records.

Preoperative Data

This included patient age, sex, smoking history, comorbidities (hypertension, diabetes mellitus, hyperlipidemia, arrhythmia, etc.), lactate value, laboratory values, neutrophil/lymphocyte ratio, monocyte/HDL ratio, drugs (beta blocker, antiarrhythmic, statin, angiotensin-converting enzyme inhibitors (ACEi), and angiotensin-receptor blockers (ARBs)), left ventricular (LV) ejection fraction (EF) value, previous cardiac or thoracic surgery, previous myocardial infarction, usage of cardiac pacemaker/intraaortic balloon pump (IABP) or extracorporeal membrane oxygenation (ECMO), type of surgery (coronary artery bypass grafting, VHS, etc.), emergency or elective surgery

Intraoperative Data

This included duration of cardiopulmonary bypass (CPB) and cross-clamp, type and amount of blood and blood products that were replaced, usage of cardiac pacemaker/IABP/ECMO, number of grafts anastomosed, type and number of replaced valves, postoperative period data, type and amount of blood and blood products that were replaced in 24 hours, usage of pacemaker/IABP/ECMO, presence of cardiac arrest/arrhythmia/myocardial infarction, type and dose of vasopressor or inotropic therapies, vasoactive-inotropic score (VIS).

Postoperative Data

This included data such as bleeding, revision surgery, cerebrovacular disease, acute kidney injury (AKI), need for renal replacement therapy (RRT), gastrointestinal system bleeding, sternal infection, sepsis, pneumonia, pleural effusion, presence of acute respiratory distress (ARDS), Acute Physiology and Chronic Health Evaluation System (APACHE II) score, Sequential Organ Failure Assessment (SOFA) score, Glasgow Coma Score (GCS), Congestive Heart Failure Classification of New York Heart Association (NYHA), European System for Cardiac Operative Risk Evaluation (EuroSCORE) score, critically patient nutritional risk score (NUTRIC), postoperative extubation time, vital signs at ICU admission, arterial blood gas analysis, mode of mechanical ventilation, ventilation parameters (tidal volume, positive end-expiratory pressure (PEEP), and fraction of inspired oxygen (FIO^2^)), arterial partial pressure of oxygen (PaO^2^), PaO^2^/FIO^2^ ratio, lactate value, reintubation, need of tracheotomy, laboratory values, neutrophil/lymphocyte ratio, C-reactive protein (CRP)/albumin ratio, Glasgow Prognostic Score (GPS), length of ICU-hospital stay, and ICU-hospital mortality (30 days).

Laboratory procedures

Laboratory examinations were complete blood count, D-dimer, coagulation profile, serum biochemical tests (glucose, renal and liver function tests, creatinine kinase, lactate dehydrogenase (LDH), and electrolytes), triglyceride, low-density lipoprotein (LDL) cholesterol, high-density lipoprotein (HDL) cholesterol, myocardial enzymes (creatine kinase, troponin), CRP, and procalcitonin (PCT). All patients underwent posterior-anterior chest radiography. The preoperative laboratory data which was used was received in preoperative anesthesia consultation. Postoperative laboratory values were within the first 24 hours after ICU admission.

The GPS score was calculated as follows; patients with an increased CRP level (>1.0 mg/dL) and low albumin level (<3.5 g/dl) were assigned a GPS of 2. Patients with only one of these biochemical abnormalities were allocated a GPS of 1. Patients with neither of these abnormalities were assigned a GPS of 0 [10].

Vasopressor and inotropic support doses were recorded postoperatively during the first 24 hours after OHS. The doses of vasopressor at ICU admission, six, 12, and 24 hours were recorded. VIS was calculated as dopamine dose (µg/kg/min) + dobutamine dose (µg/kg/min) + 100 x adrenaline dose (µg/ kg/ min) + 100 x noradrenaline dose (µg/kg/min) + 10 x milrinone dose (µg/kg/min) + 10.000 x vasopressin dose (U/kg/min) [11].

Patients younger than 75 years, who underwent aortic surgery and/or heart transplantation, with congenital heart disease, and who received right-left ventricular assist devices were excluded from the study.

Statistical analysis

The statistical analysis was performed using IBM SPSS Statistics for Windows, Version 25.0 (Released 2017; IBM Corp., Armonk, New York, United States). Frequencies were expressed as numbers (n) and percentages (%). Variables are expressed as mean values ± standard deviation. Categorical variables between the two groups were analyzed with the Chi-square test. The non-parametric continuous variables were compared by the independent samples t-test, the Mann-Whitney test for quantitative data analysis, and the Chi-square test and Fisher’s exact test were used for qualitative data analysis. Factors affecting prolonged ICU stay were determined by univariate and multivariate logistic regression analyses. p< 0.05 was considered statistically significant.

## Results

During the study period, OHS was performed on 1994 patients, 209 of which were ≥75 years. From patients who underwent aortic surgery, those with missing data were excluded. Of the 198 patients included in the study, 130 (65.7%) were male and the mean age was 78.8±3.1 years (between 75-89). Seventy patients (35.4%) had prolonged ICU stay and the length of ICU stay was <5 days in 128 patients (64.6%). CABG surgery was performed in 160 (80.8%) patients, VHS in 14 (7.1%) patients, and combined CABG and VHS in 24 (12.1%) patients. According to EuroSCORE, 102 patients (51.5%) had low risk, 56 (28.3%) medium risk, and 37 (18.7%) high risk. The patients with high EuroSCORE risk had prolonged ICU stay (30.4% vs. 12.7%, p=0.004). While the number of NYHA class I and II group patients was less in patients with prolonged ICU stay, the number of class III group patients (31.9% vs. 11.9%, p=0.011) was higher than the other group (Table 1).

**Table 1:** Demographic and Clinical Characteristics of The Patients p<0.05 was considered statistically significant SD: Standard Deviation, CABG: Coronary Artery Bypass Graft, VHS: Valvular Heart Surgery, NYHA: New York Heart Association, EuroSCORE: European System for Cardiac Operative Risk Evaluation

Variables	Number (n)/Percent (%)			
	Total (n=198)	< 5 days (n=128)	≥ 5 days (n=70)	p-value
Age, years, mean ±SD	78.8±3.1	78.1±2.7	79.9±3.5	<0.001
Body Mass Index (kg/m^2^)	26.7±4.2	27.0±4.4	26.0±3.8	0.3
Sex				0.1
Male	130 (65.7)	40 (31.3)	28 (40)	
Female	68 (34.3)	88 (68.7)	42 (60)	
Presence of smoking history	39 (20.2)	26 (21.0)	13 (18.8)	0.556
Types of Surgery				0.2
Coronary Artery Bypass Graft	160 (80.8)	112 (87.5)	48 (68.6)	
Valvular Heart Surgery	14 (7.1)	7 (5.5)	7 (10.0)	
CABG+ VHS	24 (12.1)	9 (7.0)	15 (21.4)	
Emergency of Surgery	33 (16.7)	16 (12.5)	17 (24.3)	0.04
Comorbidities				
Hypertension	145 (73.2)	96 (75.0)	49 (70.0)	0.675
Diabetes Mellitus	79 (39.9)	53 (41.4)	26 (37.1)	0.833
Cardiovascular disease	183 (92.4)	120 (93.8)	63 (90.0)	0.402
Atrial Fibrilation	46 (23.2)	26 (20.3)	20 (28.6)	0.457
Obstructive pulmonary diseases	48 (24.2)	30 (23.4)	18 (25.7)	0.992
Pulmonary hypertension	52 (26.3)	41 (32.0)	11 (15.7)	0.017
Cerebrovascular disease	28 (14.1)	18 (14.1)	10 (14.3)	0.724
Renal disease	35 (17.7)	23 (18.0)	12 (17.1)	1.000
History of Previous Thorax Surgery	10 (5.1)	3 (2.3)	7 (10.0)	0.03
History of Myocardial Infarction	67 (33.8)	41 (32.0)	26 (37.1)	0.621
NYHA Functional Classification				0.011
Class I	65 (33.3)	46 (36.5)	19 (27.5)	
Class II	84 (43.1)	59 (46.8)	25 (36.2)	
Class III	37 (19.0)	15 (11.9)	22 (31.9)	
Class IV	9 (4.6)	6 (4.8)	3 (4.3)	
EuroSCORE				0.004
Low risk	102 (52.3)	73 (57.9)	29 (42.0)	
Mild risk	56 (28.7)	37 (29.4)	19 (27.5)	
High risk	37 (19.0)	16 (12.7)	21 (30.4)	.

In patients with prolonged ICU stay, the mean age (79.9±3.5 years vs. 78.1±2.7 years p<0.001) was higher than in patients without prolonged ICU stay. The history of previous thoracic surgery (10% vs.2.3%, p=0.03) and emergency surgery (24.5% vs. 2.5%, p=0.04) were higher in patients with prolonged ICU stay compared to patients without prolonged ICU stay (Table 1). There was no statistical difference between the groups in terms of smoking, comorbidities, and surgery type (Table 1). The ICU severity scores of the patients were presented in Table 2.

**Table 2:** Intensive Care Unit Severity Scores of the Elderly Patients p<0.05 was considered statistically significant SD: Standard Deviation, APACHE: Acute Physiology and Chronic Health Evaluation, SOFA: Sepsis-Related Organ Failure Assessment, GCS: Glasgow Coma Scale, NUTRIC: The Nutrition Risk in Critically Ill

Scores	Mean±SD			
	Total (n=198)	< 5 days (n=128)	≥ 5 days (n=70)	p-value
APACHE-II	10.4±3.0	10.2 ±3.0	10.7±2.9	0.1
SOFA	5.4±2.5	5.2±2.5	5.6±2.3	0.3
GCS	14.9±0.2	14.9±0.1	14.9±0.3	0.03
NUTRIC	4.3±0.9	4.3±0.9	4.4±0.9	0.3

The patients who used statins and ACEi/ARBs in the preoperative period had a shorter ICU stay compared to those who did not (45% vs 31.4%, p=0.04; 57% vs 42.9%, p=0.03, respectively). Preoperative pacemaker usage (7% vs 0.8 %, p=0.01) was higher and preoperative EF (47.7±11.3 % vs 51.1±8.8 %, p<0.001) was lower in patients with prolonged ICU stay compared to patients without prolonged ICU stay. There was no statistical difference between the groups in terms of usage of preoperative drugs (such as beta-blockers, spironolactone, and amiodarone) and the need for RRT (Table 3).

**Table 3:** Preoperative Characteristics of the Patients p<0.05 was considered statistically significant ACEi: Angiotensin-Converting Enzyme Inhibitors, ARBs: Angiotensin Receptor Blockers, RRT: Renal Replacement Therapy, IABP: Intra-Aortic Balloon Pump, SD: Standard Deviation

Variables	Number (n)/Percent (%)			
	Total (n:198)	< 5 days (n:128)	≥ 5 days (n:70)	p-value
Types of Drugs Used				
Beta Blockers	109 (55.1)	72 (56.3)	37 (52.9)	0.657
Spironolactone	13 (6.6)0	7 (5.5)	6 (8.6)	0.389
Diuretic drugs	78 (39.4)	47 (36.7)	31 (44.3)	0.362
Amiodarone	5 (2.5)	3 (2.3)	2 (2.9)	1.00
Statins	79 (39.9)	57 (44.5)	22 (31.4)	0.04
ACEi/ARBs	103 (52.0)	73 (57.0)	30 (42.9)	0.03
Acetylsalicylic acid	103 (52.0)	67 (52.3)	36 (51.4)	0.744
Clopidogrel	33 (16.7)	23 (18.0)	10 (14.3)	0.555
RRT	7 (3.5)	5 (3.9)	2 (2.9)	1.000
Usage of Pacemaker	6 (3.0)	1 (0.8)	5 (7.2)	0.01
Usage of IABP	7 (3.5)	3 (2.3)	4 (5.7)	0.246
Ejection Fraction (%) (mean±SD)	49.9±9.9	51.1±8.8	47.7±11.3	0.01

The number of replaced valves (0.4±0.7 vs. 0.2±0.5 p=0.004) and the intraoperative last central venous pressure (CVP) were higher (14.5±4.2 vs. 13.1±4.2 p=0.03) in patients with prolonged ICU stay compared to the patients without prolonged ICU stay (Table 4).

**Table 4:** Intraoperative Characteristics of the Patients p<0.05 was considered statistically significant SD: Standard Deviation, CPB: Cardiopulmonary Bypass, RBC: Red Blood Cell, FFP: Fresh Frozen Plasma, PS: Platelet Suspension, CVP: Central Venous Pressure, IABP: Intra-Aortic Balloon Pump, ECMO: Extracorporeal Membrane Oxygenation

Variables	Mean ± SD			
	Total (n:198)	< 5 days (n:128)	≥ 5 days (n:70)	p-value
CPB Time (minute)	107.9±56.1	104.7±57.5	114.0±53.2	0.7
Aortic Clamping Time (minute)	53.9±33.0	53.4±30.1	54.9±38.0	0.3
Number of Vascular Anastomosis	3.9±3.5	4.2±4.1	3.5±1.7	0.2
Number of Replacement Valves	0.2±0.6	0.2±0.5	0.4±0.7	0.004
RBC Transfusion (unit)	2.1±1.0	2.0±1.0	2.1±0.9	0.3
FFP Transfusion (unit)	1.8±0.9	1.8±0.9	1.9±0.9	0.2
PS Transfusion (unit)	0.04±0.3	0.03±0.2	0.04±0.4	0.6
Glucose (mg/dl)	163.4±42.1	160.1±29.7	169.4±58.3	0.6
The last CVP (mmHg)	13.5±4.2	13.1±4.2	14.5±4.2	0.03
The last lactate (mmol/L)	2.4±1.3	2.3±1.3	2.6±1.3	0.2
	Number (n)/Percent (%)			
On-pump	178 (90.8)	115 (90.6)	63 (91.3)	1.0
Presence of complication	11 (5.6)	5 (3.9)	6 (8.7)	0.2
Usage of Pacemaker	3 (1.5)	1 (0.8)	2 (2.9)	0.3
Usage of IABP	9 (4.5)	3 (2.3)	6 (8.6)	0.07
Usage of ECMO	1 (0.5)	1 (0.8)	0 (0.0)	1.0

Postoperative VIS in the first 24 hours (5.6 ±8.5 vs.3.0±6.4, p<0.001), incidence of cardiac arrest (15.7% vs. 3.9%, p=0.006), arrhythmia (41.6% vs. 16.4%, p<0.001), frequency of pacemaker and IABP usage (10% vs. 0%, p=0.002; 8.6% vs. 1.6%, p=0.02), need for RRT (12.9% vs.%3.1, p=0.02), re-intubation (20% vs.1.6%,p<0.001), and tracheotomy (11.4% vs.0.8%, p=0.001) were higher in patients with prolonged ICU stay compared to patients without prolonged ICU stay. Cerebrovascular disease, delirium, sepsis, and pneumonia were observed more frequently in patients with prolonged ICU stay than patients without prolonged ICU stay and EF was higher (49.8±9.8 % vs. 46.0±10.7, p=0.01) in the group who stay <5 days in the ICU than the other group. In patients with prolonged ICU stay, while the duration of mechanical ventilation was longer (57.8±155.8 hours vs. 4.7±14.6, p<0.001), the time from ICU discharge to ICU re-admission was shorter (1.2±4.2 days vs. 1.7±13.4, p=0.006) compared to patients without prolonged ICU stay. The incidence of ICU re-admission was higher (20% vs. 2.3%, p<0.001) in patients with prolonged ICU stay than the patients without prolonged ICU stay (Table 5).

**Table 5:** Postoperative Characteristics of the Patients p<0.05 was considered statistically significant SD: Standard Deviation, RBC: Red Blood Cell, FFP: Fresh Frozen Plasma, PS: Platelet Suspension, MV: Mechanical Ventilation, ICU: Intensive Care Unit, IABP: Intra-Aortic Balloon Pump, ECMO: Extracorporeal Membrane Oxygenation, PCA: Percutaneous Coronary Angiography

Variables	Mean ± SD			
	Total (n:198)	< 5 days (n:128)	≥ 5 days (n:70)	p-value
RBC Transfusion for first 24 hours (unit)	0.4±1.1	0.4±1.2	0.5±1.0	0.14
FFP Transfusion for first 24 hours (unit)	1.7±7.1	1.8±8.8	1.5±1.5	0.05
Bleeding for first 24 hours (ml)	629.7±381.6	647.1±396.9	597.3±351.7	0.5
Vasoactive inotrope score in the first 24 hours	3.9 ± 7.3	3.0 ± 6.4	5.6 ± 8.5	<0.001
Ejection Fraction (%)	48.4±10.3	49.8±9.8	46.0±10.7	0.01
Duration of MV (hours)	23.2±95.7	4.7±14.6	57.8±155.8	<0.001
Time between ICU admission and Tracheotomy (days)	10.4±8.2	2.0±0	11.8±7.7	0.1
Time between ICU discharge and Re-admission (days)	1.5±10.4	1.7±13.4	1.2±4.2	0.006
Complications Number (n)/Percent (%)				
Cardiac Arrest	16 (8.1)	5 (3.9)	11 (15.7)	0.006
Myocardial Infarction	1 (0.5)	1(0.8)	0 (0.0)	0.4
Arrhythmia	50 (25.3)	21 (16.4)	29 (41.6)	<0.001
Usage of Pacemaker	7 (3.5)	0 (0.0)	7 (10.0)	0.002
Usage of IABP	8 (4.0)	2 (1.6)	6 (8.6)	0.02
Usage of ECMO	1 (0.5)	0 (0.0)	1 (1.4)	0.4
Revision Surgery	9 (4.5)	5 (3.9)	4 (5.7)	0.4
Need of PCA	2 (1.0)	1 (0.8)	1 (1.4)	0.2
Re-intubation	16 (8.1)	2 (1.6)	14 (20.0)	<0.001
Tracheotomy	9 (4.5)	1 (0.8)	8 (11.4)	0.001
Cerebrovascular Disease	15 (7.6)	5 (3.9)	10 (14.5)	0.01
Delirium	8 (4.0)	3 (2.3)	5 (7.1)	0.03
Acute Kidney Injury	52 (26.3)	29 (22.7)	23 (32.9)	0.3
Renal Replacement Therapy	12 (6.1)	4 (3.1)	8 (11.4)	0.02
Sternum Infeciton	5 (2.5)	3 (2.3)	2 (2.9)	0.1
Sepsis	6 (3.0)	1 (0.8)	5 (7.1)	0.04
Pneumonia	17 (8.6)	3(2.3)	14 (20.0)	<0.001
Pleural effusion	16 (8.1)	7 (5.5)	9 (12.9)	0.1
Acute Respiratory Distress Syndrome	8 (4.0)	3 82.3)	5 (7.1)	0.1
ICU re-administiration	17 (8.6)	3 (2.3)	14 (20.0)	<0.001

Preoperative hemoglobin level (11.8±1.9 vs.12.9±1.6 mg/dl, p <0.001) and hematocrit (36.5±4.8 vs. 39.1±4.8, p<0.001) were lower in patients with prolonged ICU stay compared to patients without prolonged ICU stay. Platelet level (p=0.04), CRP/albumin ratio (p<0.005), and GPS (p<0.007) were higher in patients with prolonged ICU stay than in patients without prolonged ICU stay (Table 6).

**Table 6:** Preoperative and Postoperative Laboratory Values of the Patients SD: Standard Deviation, CRP: C-reactive protein

Variables	Mean ± SD			
	Total (n:198)	< 5 days (n:128)	≥ 5 days (n:70)	p-value
Preoperative Values				
Hemoglobin (g/dl)	12.5±1.8	12.9±1.6	11.8±1.9	<0.001
Hematocrit (%)	38.2±5.0	39.1±4.8	36.5±4.8	<0.001
Platelet (10^9^/µl)	223.7±75.1	214.6±60.2	240.3±94.8	0.04
White Blood Cell (10^3^/µl)	7.5±2.4	7.4±2.5	7.7 ±2.3	0.3
Blood Urea Nitrogen (mg/dl)	22.3±9.0	22.3±9.2	22.4±8.6	0.6
Creatinine (mg/dl)	1.3±0.8	1.3±0.9	1.2±0.7	0.8
Sodium (mg/dl)	137±3.4	137.2±3.6	136.8±3.1	0.2
Potassium (mg/dl)	4.5±2.8	4.7±3.5	4.2±.0.5	0.009
Aspartate Aminotransferase (U/L)	23.0±15.1	23.0±16.2	23.2±12.5	0.5
Alanine Aminotransferase (U/L)	19.6±14.7	18.8±12.9	21.2±17.8	0.7
C-reactive Protein (mg/L)	15.2±25.5	12.9±20.7	19.3±32.1	0.01
Glucose (mg/dl)	148.9±60.1	147.9±58.2	150.4±63.9	0.9
Neutrophil-to-Lymphocyte Ratio	3.1±3.7	3.0±3.7	3.4±3.6	0.4
CRP-to-Albumin Ratio	4.8±11.2	4.5±12.1	5.2±9.4	0.005
Glasgow Prognostic Score	0.5±0.7	0.4±0.6	0.7±0.8	0.007
Postoperative Values				
Hemoglobin (g/dl)	10.7±1.1	10.7±1.1	10.6±1.1	0.7
Hematocrit (%)	31.9±3.2	31.9±3.2	32.1±3.2	0.7
Platelet (10^3^/µl)	145.2±48.3	143.4±41.2	148.5±59.3	0.9
White Blood Cell (10^3^/µl)	11.8±3.6	11.5±3.3	12.3±4.1	0.3
Blood Urea Nitrogen (mg/dl)	21.8±8.5	21.5±8.8	22.6±8.0	0.2
Creatinine (mg/dl)	1.3±0.7	1.3±0.9	1.3±0.5	0.08
Sodium (mg/dl)	139.9±3.5	140.1±3.4	139.6±3.5	0.6
Potassium (mg/dl)	4.8±8.1	5.3±10.1	4.1±0.5	0.8
Magnesium (mg/dl)	2.4±0.4	2.4±0.5	2.4±0.3	0.4
Albumin (g/dL)	3.2±0.3	3.2±0.3	3.1±0.3	0.3
Alanine Aminotransferase (U/L)	25.7±21.1	24.4±16.1	28.1±28.1	0.9
C-reactive Protein (mg/L)	53.2±31.9	53.0±30.5	53.5±34.7	0.7
Neutrophil-to-Lymphocyte Ratio	22.7±14.1	21.8±12.7	24.4±16.2	0.4
CRP-to-Albumin Ratio	17.6±13.1	17.8±14.0	17.2±11.3	0.7
Glasgow Prognostic Score	1.8±0.4	1.8±0.5	1.9±0.3	0.2

The ICU, 30-day mortality, and hospital mortality were higher in patients with prolonged ICU stay compared to patients without prolonged ICU stay (p=0.001, p=0.03, p=0.003, respectively) (Table 7).

**Table 7:** Length of ICU and Hospital Stay and Mortality of The Patients SD: Standard Deviation, ICU: Intensive Care Unit

	Total (n:198)	<5 days (n:128)	≥ 5 days (n:70)	p-value
Length of Stay (days) Mean ± SD				
ICU stay (Min-Max)	6.6±12.5 (1-152)	2.8±0.8 (1-4)	13.4±19.3 (5-152)	<0.001
Hospital stay (Min-Max)	14.5±13.4 (2-156)	10.7±.4.5 (2-33)	21.5±20.1 (8-156)	0.3
Mortality Rates N (%)				
ICU mortality	19 (9.6)	5 (3.9)	14 (20.0)	0.001
30-day mortality	14 (7.1)	5 (3.9)	9 (12.9)	0.03
Hospital mortality	30 (15.4)	12 (9.4)	18 (25.7)	0.003

According to the logistic regression analysis, higher age (OR: 1.225, 95%CI 1.104-1.360, p<0.001), preoperative pacemaker usage (OR: 0.100, 95%CI 0.01-0.969, p<0.04), preoperative statin non-usage (OR: 2.056, 95%CI 1.040-4.066, p<0.03), and preoperative low EF (OR: 0.947, 95%CI 0.915-0.981, p=0.002) were determined as independent risk factors for prolonged ICU stay (Table 8).

**Table 8:** Logistic Regression Analysis of the Effect of Risk Factors on Length of ICU Stay ICU: Intensive Care Unit, CI: Confidence Interval, ICD: Implantable Cardioverter Defibrillator, EF: Ejection Fraction

Risk Factors	Odds Ratio	95%CI	p-value
Higher Age (years)	1.225	1.104-1.360	<0.001
Non-usage of Statins Preoperatively	2.056	1.040-4.066	0.03
Usage of Preoperative Pacemaker/ICD	0.1	0.01-0.969	0.047
Preoperative low EF (<50%)	0.947	0.915-0.981	0.002

## Discussion

In our cohort study, the incidence of prolonged ICU stay was 35.4% and ICU mortality was 9.6% in patients ≥ 75 years who underwent OHS. Higher age, preoperative pacemaker/implantable cardioverter defibrillator (ICD) usage, preoperative statin non-use, and preoperative low EF (<50%) were independent risk factors for prolonged ICU stay.

In previous studies, the incidence of prolonged ICU stay among adults who underwent cardiac surgery was between 3.5% and 45%; prolonged ICU stay is considered as a wide range from 48 hours to 10 days [3-5]. In our study, the incidence of prolonged ICU stay (≥ 5 days) among patients over 75 years of age was 35.4%, which was similar to the previous studies. However, in these studies, age groups are different and the length of ICU stay is different. Therefore, the range for the incidence of prolonged ICU stay is wide. Our incidence is within this range, but it is not close to the lower limit due to the difference in our age group and prolonged ICU stay.

Similar to previously published data, one of the most important predictors was advanced age in this study [5,12-14]. In our study, the mean age was higher in patients with prolonged ICU stay than in patients without prolonged ICU stay. Advanced age and concomitant comorbidities facilitate the development of organ failure. Organ functional reserve will decrease as a process of natural aging in elderly patients. Thus, even less severe acute diseases can lead to organ failure due to decreased organ functional reserve. Elderly patients may need organ support after an acute event [15,16]. Thus, the length of ICU or hospital stay may prolong in elderly patients after cardiac surgery.

The EuroSCORE classification is predictive of postoperative complications and length of hospital stay after cardiac surgery. According to EuroSCORE, patients are classified as high risk (≥6 points), moderate risk (3-5 points), and low risk (0-2 points) [14]. In previous studies, the NYHA score was generally 3-4 and the EuroSCORE was high-risk in groups with prolonged ICU stay after cardiac surgery [2,5,13,17]. Our scores were similar to the studies. We think that patients in the prolonged ICU stay group are more at risk and need longer ICU follow-up than the other group.

In survivors of acute myocardial infarction with asymptomatic LV dysfunction (LV EF <35-40%), randomized controlled trials have shown that ACEi reduced mortality, hospitalizations, and progression to severe heart failure compared with placebo [18,19]. In individuals with asymptomatic LV dysfunction, enalapril has been associated with reduced heart failure hospitalization and mortality compared to placebo [19,20]. In the Cooperative North Scandinavian Enalapril Survival Study (CONSENSUS) trial (enalapril vs. placebo), where the mean age (71 years) was close to our study, mortality was significantly reduced in the enalapril arm (26% vs 44% after six months) [21]. A meta-analysis with a mean age of 75.1 presented that ACEi as monotherapy or first-line therapy reduces heart failure-related rehospitalization and all-cause mortality in heart failure with preserved EF [22]. In our cohort, the patients who used ACEi/ARBs in the preoperative period had a shorter ICU stay compared to those who did not. Thus, we thought that ACEi/ARBs used preoperatively may improve cardiac function and so, it may shorten the length of ICU and/or hospital stay after cardiac surgery.

In our cohort, the history of previous thoracic surgery and emergency surgery was higher in patients with prolonged ICU stay compared to patients without prolonged ICU stay. Previous studies presented that previous cardiac surgery and emergency operations contributed to extended ICU stays [23-25]. Previous thoracic surgeries may change thoracic anatomy, cause adhesions in the skin and subcutaneous tissue, impair chest wall compliance, and predispose to bleeding. In emergency surgeries, hemodynamic instability occurs. These reasons may cause respiratory and cardiac complications in the postoperative management of patients after cardiac surgery [2,26,27]. Thus, in these patients, the length of ICU stay may be extended.

The number of replaced valves and the intraoperative last CVP were higher in patients with prolonged ICU stay compared to patients without prolonged ICU stay. Previous studies have shown that multiple valves replacement are associated with an increased incidence of major complications and hospital mortality [28,29]. CPB time and aortic clamping time are prolonged in patients who undergo multiple valves replacement surgery. Prolonged CPB and aortic clamping times are associated with prolonged ICU stay [30]. In our study, concomitant multiple valves replacement and higher incidence of comorbidity associated with advanced age prolonged the length of ICU stay. Venous congestion due to high CVP during perioperative cardiac surgery is associated with poor patient outcomes and prolonged ICU/hospital stays [30,31]. High CVP impedes venous return and causes multiple organ dysfunction. Organs need time to recover their functions. This condition may prolong the length of ICU and/or hospital stay.

Preoperative usage of pacemaker/ICD was an independent risk factor for prolonged ICU stay in our study. Patients with a new/old pacemaker and/or ICD device in the preoperative period have arrhythmia problems. Arrhythmias are very common complications after cardiac surgery and represent a major source of morbidity and mortality and are associated with prolonged ICU and/or hospital stay [32]. In previous studies, patients with preoperative arrhythmia had longer ICU and/or hospital stay than those without preoperative arrhythmia [4,33,34]. Bradycardia and complete atrioventricular block are also types of arrhythmia. Although pacemaker and/or ICD correct these arrhythmias, patients with a pacemaker/ICD in the preoperative period are at risk for prolonged ICU stay due to difficulties of postoperative management. Thus, the length of ICU/ hospital stay of the patients may be prolonged.

Kuhn et al. reported that statin therapy was associated with a shorter ICU and hospital stay for patients undergoing cardiac surgery [35]. High cholesterol levels have been associated with the presence of cardiovascular disease. Statins (hydroxymethylglutaryl-coenzyme A (HMG-CoA) reductase inhibitors) decrease blood cholesterol levels, improve endothelial function, stabilize plaque, reduce inflammatory markers, and prevent myocardial ischemia-reperfusion injury [35,36]. Statins also increase endothelial nitric oxide (NO) production, inhibit the expression of proinflammatory mediators, reduce myocardial damage, and are effective to reduce mortality and morbidity in patients with cardiovascular disease. Previous studies have shown that statins may protect direct organs and improve postoperative outcomes in patients undergoing cardiac surgery [35,37]. In addition, statins are expected to reduce the incidence of both cardiac and non-cardiac events. Randomized controlled trials reported that preprocedural statin therapy reduced periprocedural cardiovascular events in patients who have undergone percutaneous coronary intervention and non-cardiac surgery [38,39]. Thus, it is associated with lower mortality in patients with atherosclerotic cardiovascular disease. In addition, similar to all data, we found that not using preoperative statins was an independent risk factor for prolonged ICU stay.

Preoperative low EF (<50%) has been identified as the independent risk factor for prolonged ICU stay in our study. Tunç et al. reported that low EF was a significant risk factor for > 48 hours of ICU stay [4]. In many studies, preoperative EF was found to be lower in the group with prolonged ICU and/or mechanical ventilation stay after cardiac surgery than the group without prolonged ICU stay [4,5,25,33]. Silberman et al. showed that preoperative LV dysfunction was more common in the group with ICU stay >14 days after cardiac surgery than the group without [34]. Atoui et al. and Wang et al. presented that one of the independent predictors for extended ICU stay was a low EF [40,41]. Low EF causes postoperative low cardiac output syndrome with decreased organ perfusion [19,42]. Thus, hemodynamic instability, acute kidney injury, respiratory failure, atrial fibrillation, stroke, sepsis, endocarditis, deep sternal wound infection, bleeding requiring reoperation, and gastrointestinal bleeding are seen. Respiratory, cardiac, and renal organ support may be needed [42]. A heart with preoperative low cardiac reserve regains normal cardiac function in the postoperative late period. Therefore, this condition may extend the length of ICU stay.

Several studies also showed that postoperative low EF is correlated with prolonged ICU/hospital stay [4,43]. In our study, EF was higher in the group who stay <5 days in the ICU when compared to the other group. Since the cardiac function of patients with high EF is good, the need for ICU and close follow-up may decrease. Thus, the length of ICU stay may be shorter.

In our study, the patients with prolonged ICU stay had more postoperative complications -such as cardiac arrest, hemodynamic instability, arrhythmia, pacemaker and IABP usage, RRT, re-intubation, tracheotomy, cerebrovascular disease, delirium, sepsis, and pneumonia. Previous studies presented that postoperative complications are higher in those with prolonged ICU and/or hospital stay [3-5,34]. Patients with complications after cardiac surgery need ICU follow-up and management. For these reasons, the expected result is that these patients may require longer ICU stay. On the other hand, some events, such as sepsis, may be the result, not the cause, of prolonged ICU stay.

Many clinical trials showed that preoperative anemia is associated with adverse outcomes, morbidity, mortality, and length of ICU/hospital stay after cardiac surgery [44-46]. Anemia decreases oxygen delivery to tissues, and end-organ dysfunction can ensue, particularly in patients with atherosclerotic disease [46]. Our patients with prolonged ICU stay had lower preoperative hemoglobin levels and hematocrit. We think that we may shorten the length of ICU and/or hospital stay by treating preoperative anemia.

The GPS includes the value of albumin and CRP, which are associated with inflammation and malnutrition [47,48]. Several studies have presented that inflammation plays an important role in the pathogenesis of numerous cardiovascular diseases from endothelial damage to atherosclerotic plaque formation and plaque rupture in coronary artery diseases, disease progression in heart failure, and the development of arrhythmias [47,49]. In our study, we found that the preoperative CRP albumin ratio and GPS were higher in patients with a prolonged ICU stay. The complications, length of ICU stay, and mortality in these patients may be shorter by appropriate nutritional protocols and administering medications that will reduce inflammation preoperatively.

Tribuddharat et al. reported that platelet counts ≤120×10^3^/mm were associated with prolonged ICU stay in a study that included patients aged between 18 and 75 years [50]. While we found higher platelet counts in the group with prolonged ICU stay, our patients were >75 years old. The reason may be that inflammatory responses are different during every stage of advanced age [50].

Patients with prolonged ICU stay during the postoperative period are those who were more at risk in the preoperative, intraoperative, and postoperative periods. These high-risk patients may be readmitted to the ICU multiple times and in a short time after ICU discharge [51,52]. In the group with prolonged ICU stay, the incidence of ICU re-admission was higher and the time from ICU discharge to ICU re-admission was shorter compared to the other group similar to previous studies.

This study has some limitations. It was a retrospective study. It was conducted at a single center, which limits the generalizability of the results. The data were collected from the digital patient records.

## Conclusions

In our study, the predictors for prolonged ICU stay included higher age, preoperative pacemaker usage, preoperative statin non-use, and low preoperative EF. The incidence of prolonged ICU stay among patients ≥75 years after OHS was 35.4%. Identifying risk factors associated with prolonged ICU stay among elderly patients undergoing cardiac surgery may help to reduce and prevent risk factors. Thus, perioperative morbidity/mortality may be decreased, the length of ICU and/or hospital stay may be shortened, hospital costs may be reduced, and the quality of life of patients may be improved.
